# Role of Chaperone Mediated Autophagy (CMA) in the Degradation of Misfolded N-CoR Protein in Non-Small Cell Lung Cancer (NSCLC) Cells

**DOI:** 10.1371/journal.pone.0025268

**Published:** 2011-09-23

**Authors:** Azhar Bin Ali, Dawn Sijin Nin, John Tam, Matiullah Khan

**Affiliations:** 1 Cancer Science Institute, Yong Loo Lin School of Medicine, National University of Singapore, Singapore; 2 Department of Medicine, Yong Loo Lin School of Medicine, National University of Singapore, Singapore; 3 Department of Surgery, Yong Loo Lin School of Medicine, National University of Singapore, Singapore; 4 Departments of Cardiac, Thoracic & Vascular Surgery, National University Hospital, Singapore, Singapore; Boston University Medical School, United States of America

## Abstract

Nuclear receptor co-repressor (N-CoR) plays important role in transcriptional control mediated by several tumor suppressor proteins. Recently, we reported a role of misfolded-conformation dependent loss (MCDL) of N-CoR in the activation of oncogenic survival pathway in acute promyelocytic leukemia (APL). Since N-CoR plays important role in cellular homeostasis in various tissues, therefore, we hypothesized that an APL like MCDL of N-CoR might also be involved in other malignancy. Indeed, our initial screening of N-CoR status in various leukemia and solid tumor cells revealed an APL like MCDL of N-CoR in primary and secondary tumor cells derived from non-small cell lung cancer (NSCLC). The NSCLC cell specific N-CoR loss could be blocked by Kaletra, a clinical grade protease inhibitor and by genistein, an inhibitor of N-CoR misfolding previously characterized by us. The misfolded N-CoR presented in NSCLC cells was linked to the amplification of ER stress and was subjected to degradation by NSCLC cell specific aberrant protease activity. In NSCLC cells, misfolded N-CoR was found to be associated with Hsc70, a molecular chaperone involved in chaperone mediated autophagy (CMA). Genetic and chemical inhibition of Lamp2A, a rate limiting factor of CMA, significantly blocked the loss of N-CoR in NSCLC cells, suggesting a crucial role of CMA in N-CoR degradation. These findings identify an important role of CMA-induced degradation of misfolded N-CoR in the neutralization of ER stress and suggest a possible role of misfolded N-CoR protein in the activation of oncogenic survival pathway in NSCLC cells.

## Introduction

Transcriptional factors and their cognate co-factors are the major regulators of development of epithelial cells and are among the most frequent targets of oncogenic insult in various human malignancies including Lung cancer [1]–[4]. Transcriptional control imparted by nuclear receptor co-repressor (N-CoR) plays important role in the growth suppressive function of several tumor suppressor proteins [5], [6]. Deregulation of N-CoR mediated transcriptional control due to a misfolded conformation dependent loss (MCDL) of N-CoR has been implicated in the pathogenesis of acute promyelocytic leukemia (APL) [7], [8]. Interestingly, some of the transcription factors and co-factors that were initially identified as the regulators of normal hematopoiesis were also found to be involved in the regulation of normal growth and development of lung epithelial cells [2]–[4]. These finding suggested that considerable overlapping might also exist in the mechanism underlying the hematological malignancies and Lung Cancer. Lung cancer is the leading cause of cancer related mortality and morbidity in human worldwide. With a 5-year survival rate of only 15%, it is regarded as one of the most fatal cancers in human. Based on currently known histo-pathological criteria, lung cancer is broadly categorized into two major subtypes: non-small-cell lung cancer [9], [10] and small-cell lung cancer (SCLC) [11]. NSCLC, which comprises of 80% of all lung cancers in human, is further subdivided into adenocarcinomas, squamous cell carcinomas, adenosquamous carcinomas, and large-cell carcinomas [12]. Despite being the leading cause of human mortality and mobility, very little is known about the mechanism underlying the malignant growth and transformation of cells that form the bulk of tumor mass in lung cancer. This is partly due to a lack of clear understanding of the oncogenic survival mechanism that sustains the growth of malignant cells in nutrient depleted and stressful microenvironment widely presented within the solid mass of Lung cancer tissue.

Nuclear receptor co-repressor (N-CoR) is a vital component of a multi-protein repressor complex essential for the function of many transcription factors and tumor suppressor proteins [13]–[16]. Since transcriptional control imparted by factors like N-CoR is thought to be involved in the regulation of normal growth and development of cells in various tissue subtypes, therefore, we hypothesized that an APL like MCDL of N-CoR might also be linked to the oncogenic growth in other human tumors. To test this hypothesis, we analyzed N-CoR status in tumor cells derived from major human leukemia and solid tumors and observed a selective misfolded conformation dependent loss of N-CoR in multiple tumor cell lines and primary human cancer tissues derived from NSCLC. In NSCLC cells, misfolded N-CoR was found to be associated with Hsc70, a molecular chaperone involved in chaperone mediated autophagy (CMA). Genetic and chemical inhibition of CMA led to N-CoR stabilization in NSCLC cells, suggesting a crucial role of CMA in N-CoR loss. These findings illustrate a possible role of autophagic degradation of misfolded N-CoR in the neutralization of ER stress as well as in the survival and growth of NSCLC cells.

## Materials and Methods

### Cell lines and reagents

All the lung cancer cell lines used in this study were purchased from American Type Culture Collection (ATCC) and were maintained in medium recommended by ATCC. Genistein, Chloroquine and Nicotine were purchased from Sigma (St. Louis, MO, USA) while Kaletra was from Abbott Laboratories (IL, USA). N-CoR (C-20), PDI and Hsc-70 antibodies were purchased from Santa Cruz Biotechnology (Santa Cruz, CA, USA). Antibodies against Lamp-2A (Abcam Inc, MA, USA) and β-actin (Sigma, St Louis, MO, USA) were purchased from respective sources. Recombinant flag-tagged N-CoR was prepared from 293T cells [17] transfected with N-CoR expression plasmid (linked with two tandem flag sequence) using the Fugene 6 (Roche, Basel, Switzerland). The N-CoR and control siRNA duplex described previously [18], [19] were synthesized with minor modification in N-CoR sequence (Qiagen GmBH, Hilden, Germany), and were transfected using oligofectamine reagent (Invitrogen, Carlsbad, CA, USA).

### Primary human lung tumor samples and tissue arrays

Ten histologically confirmed primary human NSCLC samples were obtained from the tissue repository of National University Hospital-National University of Singapore (NUH-NUS) with the approval of National University of Singapore-Institutional Review Board (NUS-IRB). These samples were snap-frozen within 40 minutes (on average) of surgical resection and stored at −80°C. Human lung cancer tissue array BC04012 was purchased from Biomax (US Biomax Inc, Maryland, USA) and stained with N-CoR (C-20) antibody using ABC staining system (Santa Cruz, CA, USA) according to manufacturer guidelines.

### Semi-quantitative and real time PCR assay

Total RNA was isolated with RNeasy Mini Kit (Qiagen GmBH, Hilden, Germany). From each sample, 2 µg of RNA was converted into cDNA by oligo (dT)_18_-primed reverse transcription using SuperScript II RT First-Strand kit (Invitrogen, Carlsbad, CA, USA) as described by the manufacturer. The cDNA was subject to semi-quantitative PCR analysis using Accuprime Taq polymerase system (Invitrogen, Carlsbad, CA, USA) according to manufacturer's recommendations. The real time PCR analysis was carried out using the Taqman® Gene Expression Assay System (Applied Biosystems, CA,USA) and C_t_ values were recorded using the ABI Prism 7300 Real Time PCR system (Applied Biosystems, CA, USA).

### Analysis of real-time PCR data

Data was analyzed using the comparative C_t_ method where the cell line SAEC was used as the reference sample and the HPRT gene was used as the endogenous gene control. Data was represented in a bar graph plotted on log scale with a base of 10. N-CoR expression level in various cell lines was calculated in relative to the N-CoR level in SAEC [20] cells which was set to 0. Data represented is the average obtained from 3 independent experiments.

### In-vitro N-CoR cleavage assay

Crude cellular extracts containing active proteases were obtained by incubating, lung cancer cells or cryo-preserved human primary lung cancer tissues in RSB buffer [10 mM Tris (pH 8.0), 10 mM NaCl, 3 mM MgCl_2_, 0.1% NP-40] at 4°C for 10 min and nuclei were then removed by centrifugation. The supernatants were harvested and protein content was subsequently determined. N-CoR substrate was prepared by transfection of 293T cells with Flag-tagged N-CoR plasmid. Optimized cleavage assay was performed in 300 mM NaCl, 50 mM Tris (pH 8.0), at 37°C for various time duration. The reaction was terminated by heating at 50°C in SDS sample buffer, and proteins were resolved with SDS-PAGE and transferred to PVDF membrane for western blotting.

### Immunoprecipitation

Two approaches were designed to detect the physical interaction between N-CoR and Hsc70. In one approach, protease depleted HLPC fractionates of H2170 cells was incubated with flag-tagged N-CoR in IP buffer [40 mM Tris-HCl (pH 7.4), 150 mM NaCl, 0.5 mM EDTA, 0.75 mM MgCl_2_, 0.25% NP-40] for 5 mins with rotation at 4°C. Anti-flag M2 affinity gel beads (Sigma) were added and further incubated for 2 hours with rotation at 4°C. In second approach, vehicle or Kaletra-treated H2170 cells (at 5 µM for 96 hours) were sonicated in lysis buffer [150 mM NaCl, 0.5% NP40, 1 mM EDTA, 20 mM Tris (pH 7.4), 1 mM NaF, 1 mM Na_2_VO_4_, 20 mM β-glycerolphosphate, 1.5 mg/ml IAA, protease inhibitor cocktail] for 10 s. The lysates were cleared by centrifugation and subsequently incubated with Hsc70 or control antibodies for 2.5 hours with rotation at 4°C. Protein G beads were then added followed by 1.5 hours rotation at 4°C. Immunoprecipitates were resolved on SDS-PAGE gel and N-CoR and Hsc70 were detected by western blotting.

### Immunoflorescence staining

Cells were smeared onto glass slides using cytospin centrifugation, fixed with 4% paraformaldehyde and permeabilized using 0.2% Triton X-100 in PBS at 4°C. The fixed cells were subsequently stained with relevant primary antibodies followed by FITC- or rhodamine-conjugated secondary antibodies (Invitrogen-Molecular Probes, Carlsbad, CA, USA). DNA was stained with DAPI (Invitrogen-Molecular Probes) and fluorescence signals were captured using confocal microscopy.

### Protein solubility assay

N-CoR solubility assay was performed as described previously with some modification [8]. Briefly, cells were sonicated briefly in cell lysis buffer [50 mM Tris (pH 8.0), 5 mM MgCl_2_, 100 mM NaCl, 0.5% NP-40 and protease inhibitor cocktail] and kept on ice for 30 minutes. The soluble and insoluble fractions were separated through centrifugation at 15,000 rpm for 10 minutes at 4°C. The insoluble fraction was treated with DNAse I for 30 minutes at 37°C. The soluble and insoluble fractions were then resolved on SDS-PAGE and stained with anti-N-CoR (C-20).

### Lysosomal uptake assay

Lysosomes were isolated from H2170 cells using the Lysosome Enrichment Kit (Thermo Fisher Scientific, Waltham, MA, USA). The lysosomal uptake assay, using the flag-tagged N-CoR as substrate, was performed essentially as described elsewhere [21].

## Results

### Post-transcriptional loss of N-CoR in tumor cells derived from NSCLC

**Figure 1:**
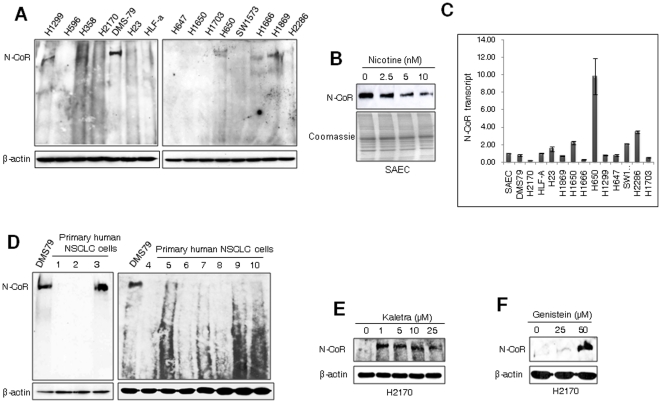
Selective loss of N-CoR protein in NSCLC cells. A & B, Level and integrity of full length N-CoR protein in various lung cancer cells was determined through western blotting assay. Level of β-actin was determined as experimental control. DMS-79 is derived from SCLC while rests of the cells are derived from NSCLC. B, Level of full length N-CoR protein in SAEC treated with nicotine for 48 hours in a dose dependent manner was determined. C, Level of N-CoR transcript in cell lines used in Figure 1 A & B was quantified by real time PCR. The identity of cells used in figure 1A & C is presented in Supporting Information S1. D, Level of full length N-CoR protein in ten histologically confirmed human primary NSCLC cells was determined in western blotting assay with N-CoR antibody. The identity of human samples is presented in Supporting Information S1. E and F, Level of intact N-CoR protein in H2170 cells treated with Kaletra (E) or genistein (F) was determined was determined in western blotting assay.

Analysis of N-CoR status in various lung cancer cells revealed an apparent loss of N-CoR at protein level in multiple tumor cell lines derived from NSCLC, but not in DMS-79; a cell line derived from SCLC (Fig. 1A, Supporting Information S1). A similar pattern of N-CoR loss was also observed in normal small airway epithelial cells (SAEC) after treatment with nicotine, the carcinogenic agent widely linked to Lung cancer (Fig. 1B). The N-CoR loss observed in NSCLC cells was most likely a post-transcriptional event since level of N-CoR transcript, as determined by real-time PCR, was not significantly down regulated when compared to its level in DMS-79 or SAEC cells (Fig. 1C). Identical pattern of N-CoR loss at protein level was also observed in nine out of ten histologically confirmed human primary NSCLC cells, suggesting that N-CoR loss was not an exclusive event limited to NSCLC cell lines (Fig. 1D, Supporting Information S1). The N-CoR loss observed in H2170 cells could be blocked by Kaletra, a clinical grade HIV protease inhibitor and a documented inhibitor of growth of NSCLC cells (Fig. 1E) [22]. N-CoR loss was also blocked by genistein (Fig. 1F), an inhibitor of N-CoR misfolding previously characterized by us [23]. These finding suggested that the loss of misfolded N-CoR could be causally linked to the growth and survival of NSCLC cells.

**Figure 2:**
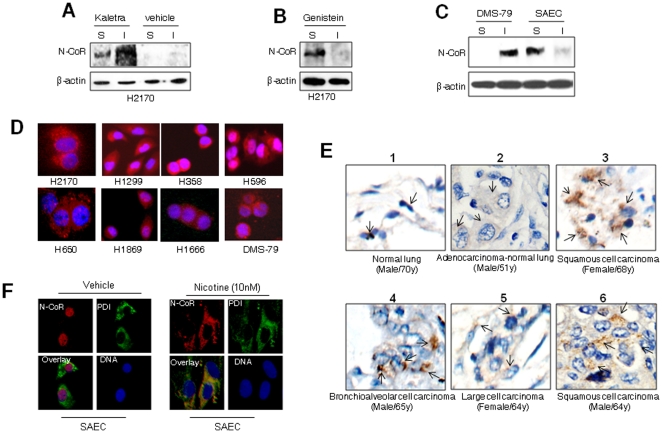
Loss of N-CoR in NSCLC cells is linked to misfolding. A, B & C, Solubility (S)/insolubility (I) of N-CoR or β-actin in Kaletra (A) or genistein (B) treated H2170 cells, as well as in DMS-79 or SAEC cells (C), was determined by protein solubility assay. D, Subcellular distribution of N-CoR (red signal) in NSCLC (H2170, H1299, H358, H596, H650, H1869 and H1666) and SCLC (DMS-79) cells was determined by immunoflorescence assay performed with N-CoR antibody. DNA (blue signal) was stained with DAPI. E, Subcellular distribution of N-CoR in histologically confirmed human primary NSCLC tissue sections (panel 2–6) and normal human lung tissue section (panel 1) was determined by immunohistochemical (IHC) assay performed with N-CoR antibody. The brownish staining in the cytosol pinpointed by black arrow represents the N-CoR signal (panels 2–6). N-CoR staining in normal lung tissue section is visible as black dots overlapping the nucleus (panel 1). The second panel demonstrates a cross section that contains both cancer tissue (left half) and normal lung tissue (right half) side by side. F, Subcellular distribution of N-CoR and PDI in SAEC cells treated with vehicle or nicotine for 48 hours was determined by confocal microscopy.

Stabilization of N-CoR by genistein suggested that NSCLC cell specific N-CoR loss was most likely triggered by its misfolding as observed previously in APL. To confirm that, conformation of N-CoR protein stabilized by Kaletra or genistein was determined. In native conformation, N-CoR is confined to the nucleus and remains soluble in buffer containing organic detergent; while in misfolded conformation, N-CoR becomes insoluble and is translocated to endoplasmic reticulum [7], [8]. We used these criteria to determine whether N-CoR that was subjected to degradation in NSCLC cells was misfolded. A significant portion of N-CoR stabilized by Kaletra was found in the insoluble fraction of H2170 cells, suggesting that H2170 cells harbored a misfolded N-CoR protein (Fig. 2A). On the other hand, N-CoR stabilized by genistein was largely detergent soluble, suggesting that genistein could rescue the native N-CoR conformation (Fig. 2B). A major portion of N-CoR in normal small airway epithelial cells (SAEC) was found in soluble fraction, while in SCLC derived cells DSM-79, N-CoR was largely insoluble (Fig. 2C). Consistent with the finding of solubility assay, N-CoR displayed a predominantly cytosolic distribution in multiple NSCLC derived cells (Fig. 2D, red signal) and histologically confirmed human primary NSCLC tissue sections (Fig. 2E, panels 2–6). In contrast, N-CoR was mainly localized in the nucleus in human normal lung epithelial cells (Fig. 2E, panel 1) and in SAEC cells (Fig. 2F, left panels). The nuclear N-CoR found in SAEC cells was translocated to ER after nicotine treatment, suggesting that nicotine can trigger N-CoR misfolding (Fig. 2F, right panels). Collectively, these finding suggested that N-CoR that was subjected to degradation in NSCLC cells was most likely a misfolded protein.

### NSCLC cell specific N-CoR loss is linked to endoplasmic reticulum (ER) stress

**Figure 3:**
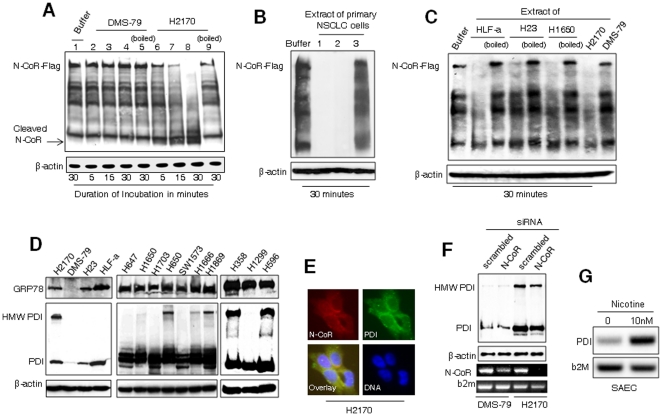
NSCLC cell specific N-CoR loss is linked to endoplasmic reticulum (ER) stress. A, H2170 cells harbor N-CoR cleaving activity. Processing of Flag-tagged N-CoR incubated with the extracts of DMS-79 (lanes 2–5) or H2170 (lanes 6–9) cells for the duration as mentioned was determined by western blotting assay performed with Flag antibody. The extract loaded in lanes labeled as “boiled” was heated at 100°C for 10 minutes prior to incubation. Equal volume of buffer lacking cell extract was used in lane labeled as “buffer”. The arrow marks position of the cleaved 100 kDa N-CoR fragment. B, Processing Flag-tagged N-CoR incubated with the extracts of first three human primary NSCLC cells (Supplementary table 2) was determined by western blotting with Flag antibody. C, N-CoR cleavage assay, using extracts of NSCLC cells HLF-a, H23 or H1650, was performed essentially as described in the legend of Figure 2A. D, Level of ER stress in various lung cancer cells was analyzed by determining the levels of GRP-78 and PDI (basal and HMW: high molecular weight). E, N-CoR localized to ER was visualized by staining H2170 cells with N-CoR (red) and PDI (green) antibodies. DNA was stained with DAPI (blue). F, Level of PDI in H2170 or DMS-79 cells exposed to scramble or N-CoR siRNA was determined by western blotting (upper panel). The N-CoR knock-down efficiency in each subset of cells was confirmed by RT-PCR assay (lower panels). G, Level of PDI transcript in SAEC cells treated with nicotine for 48 hours was determined by RT-PCR analysis.

In APL, N-CoR loss was an outcome of cytoprotective UPR which was mediated by APL cell specific aberrant protease activity that eventually protected APL cells from ER stress-induced apoptosis [7]. To investigate whether N-CoR loss in NSCLC cells was also facilitated by similar aberrant protease activity, an optimized N-CoR cleavage assay was performed. In this assay, flag-tagged N-CoR ectopically expressed in 293T cells was incubated with the extract of H2170 cells, an NSCLC derived cell line that exhibited N-CoR loss, or DMS-79 cells, a SCLC derived cell line that did not exhibit any N-CoR loss, and the level of N-CoR digested during this incubation was determined through western blotting assay. Flag-tagged N-CoR incubated with the extract of H2170 cells was digested in a time dependent manner and a cleaved N-CoR fragment of 100 kDa was generated (Fig. 3A, lanes 6–8), while N-CoR-Flag incubated with the extract of DMS-79 cells was not digested (Fig. 3A, lanes 2–4). Interestingly, the N-CoR cleaving activity presented in H2170 cells was completely deactivated by boiling, suggesting that the activity might be a heat labile protease (Fig. 3A, lane 9). As observed with NSCLC derived H2170 cells, extracts of two human primary NSCLC cells, in which no full length N-CoR was detected, contained activity that completely digested the flag-tagged N-CoR protein (Fig. 3B). Identical heat-labile N-CoR cleaving activity was also found in three other representative NSCLC cells HLF-a, H23 and H1650 (Fig. 3C).

Next to test whether NSCLC cell specific N-CoR loss was linked to ER stress as observed in APL, level of ER stress across NSCLC cells was compared to that of DMS-79 cells by measuring the levels of GRP78 and PDI proteins, two bona fide markers of ER stress [24], [25]. Levels of GRP78 and PDI, both normal and a high molecular weight (HMW) variant specifically found in cells undergoing ER stress, were significantly higher in all NSCLC cells that exhibited N-CoR loss, whereas these two proteins were almost undetectable in DMS-79 cells (Fig. 3D). A possible role of misfolded N-CoR in the amplification of ER stress was suggested by the co-localization of N-CoR and PDI in H2170 cells (Fig. 3E), and by the reduction of PDI level after N-CoR knockdown (Fig. 3F). Moreover, treatment of SAEC cells with nicotine at concentration that triggered N-CoR loss led to up regulation PDI level, suggesting that nicotine-induced N-CoR loss could also be linked to ER stress (Fig. 3G). These findings collectively suggested a link between N-CoR loss and the amplification of ER stress in NSCLC cells. It also suggested that degradation of misfolded N-CoR may have contributed to the attenuation of ER stress and eventual protection of NSCLC cells from ER stress-induced apoptosis as previously found in APL.

### Misfolded N-CoR is degraded through chaperone mediated autophagy

**Figure 4:**
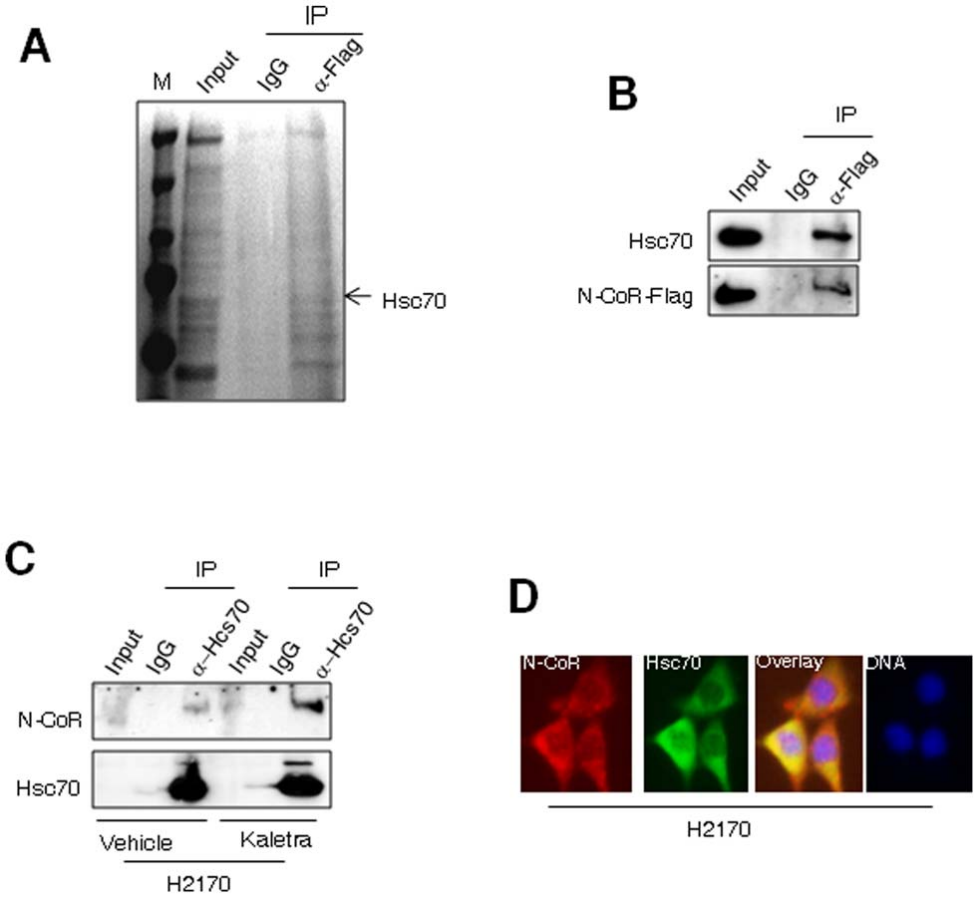
N-CoR is a substrate of chaperone mediated autophagy (CMA). A, Column purified cytosolic extracts of H2170 cells was incubated with flag-tagged N-CoR ectopically expressed in 293T cells. N-CoR was immunoprecipitated with anti-flag antibody and proteins co-precipitated with N-CoR were resolved in SDS-PAGE, excised and their identity was determined by MS. B, The association between Flag-tagged N-CoR and Hsc70 was reconfirmed in co-immunoprecipitation assay performed essentially as described in legend of figure 4A. After detection of co-precipitated Hsc70 with Hsc70 antibody (upper panel), the membrane was re-probed with flag antibody to quantify the amount of precipitated N-CoR protein (lower panel). In “input” lanes, an aliquot of whole cell extract was loaded. C, Hsc70 was immunoprecipitated from the extracts of vehicle or 5 µM Kaletra treated H2170 cells and level of co-precipitated N-CoR was determined with N-CoR antibody (upper panel). The membrane was re-probed with Hsc70 antibody (lower panel). In “input” lanes, an aliquot of whole cell extract was loaded. D, N-CoR (red) and Hsc70 (green) distribution in H2170 cells was determined through immunoflorescence analysis using confocal microscopy. The intensity of yellow signals signifies the degree of co-localization of two proteins. DNA was labeled with DAPI (blue).

To gain further insight into the mechanism underlying the loss of N-CoR and to understand the functional consequence of N-CoR loss, we decided to identify the proteins that may have associated with misfolded N-CoR protein before its degradation. To achieve this objective, a specially designed co-immunoprecipitation assay was performed using protease-depleted cytosolic extract of H2170 cells and flag-tagged N-CoR ectopically expressed in 293T cells. Flag-tagged N-CoR incubated with protease-depleted cytosolic extract of H2170 cells was immunoprecipitated with flag antibody and the identity of proteins associated with N-CoR was determined by mass spectrometry (MS) after their separation in SDS-PAGE. Interestingly, out of several proteins co-precipitated with flag-tagged N-CoR, one was identified as Hsc70 (Fig. 4A), a molecular chaperone essential for the translocation of specific CMA substrates to the lysosomes [26]–[28]. To test the direct association between N-CoR and Hsc70, an aliquot of protease-depleted cytosolic extract of H2170 cells was incubated with the extract of 293T cells transfected with flag-tagged N-CoR plasmid, and level of Hsc70 precipitated with N-CoR was determined in western blotting assay. Significant amount of Hsc70 protein (Fig. 4B, upper panel) was co-precipitated along with the Flag-tagged N-CoR (Fig. 4B, lower panel) precipitated by Flag antibody. The association between N-CoR and Hsc70 proteins was further confirmed in co-immunoprecipitation assay performed with the extracts of vehicle or Kaletra treated H2170 cells (Fig. 4C). Moreover, in indirect immunoflorescence assay, significant co-localization between N-CoR and Hsc70 was observed, further suggesting their in-vivo association in H2170 cells (Fig. 4D).

**Figure 5:**
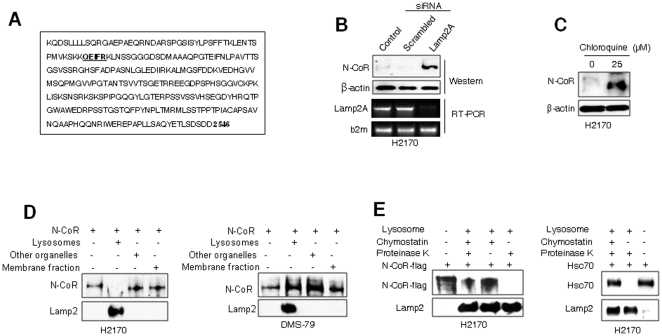
N-CoR is degraded by chaperone mediated autophagy (CMA). A, N-CoR contains a consensus lysosomal targeting motif. The lysosomal targeting motif in N-CoR peptide sequence is highlighted in underlined bold. B, Lamp2A ablation blocked N-CoR loss in H2170 cells. N-CoR level in H2170 cells exposed to anti-Lamp2A or control siRNA for 72 hours was determined with N-CoR antibody (upper panel) after confirming Lamp2A ablation by RT-PCR analysis (lower panel). C, Chemical inhibition of lysosomal function stabilized N-CoR. Level of N-CoR in H2170 cells treated for 72 hours with vehicle or 25 µM chloroquine, a selective inhibitor of lysosomal function, was determined with N-CoR antibody. D, Flag-tagged N-CoR was incubated with purified lysosomal or non-lysosomal fractions of H2170 (left) or DMS-79 (right) cells and level of N-CoR degradation was determined with flag antibody. Level of Lamp2, a lysosomal protein, was determined to demonstrate the purity of each fraction. E, N-CoR taken up by chymostatin treated lysosomes was protected from proteinase K digestion. Flag-tagged N-CoR or Hsc70 incubated with lysosomes isolated form H2170 cells pretreated with chymostatin was subjected to proteinase K digestion and relative level of protected N-CoR or Hsc70 was determined with flag or Hsc70 antibody. Lamp2 level was determined to quantify the amount of lysosomes in each sample.

Most of the known substrates of CMA contain a lysosomal targeting motif biochemically related to KFERQ [21], [29]. This motif consists of a Q flanked on either sides by four amino acids consisting of a basic, an acidic, a bulky hydrophobic, and a repeated basic or bulky hydrophobic amino acid. An analysis of N-CoR peptide sequence revealed the presence of a nearly identical lysosomal targeting motif comprising of **QEIFR** pentapeptide, suggesting that N-CoR might be a substrate of CMA (Fig. 5A). In CMA, Hsc70 first associates with its misfolded cargo in the cytosole and then Hsc70-cargo complex is transported to the lysosome [29], [30]. Once delivered to the lysosomal membrane by Hsc70, the misfolded cargo proteins are translocated to the lysosomal cavity with the help of Lamp2A, a rate limiting factor of CMA [26]–[28]. To prove that N-CoR was degraded through the Lamp2A-mediated lysosomal pathway, effect of Lamp2A ablation on the level of N-CoR protein was determined. N-CoR loss was significantly reversed (Fig. 5B, upper panels) after Lamp2A ablation with an effective anti-Lamp2A siRNA (Fig. 5B, lower panels), suggesting an important role of Lamp2A in the loss of N-CoR protein. Moreover, treatment of H2170 cells with chloroquine, a chemical inhibitor of lysosomal function [31], significantly blocked the loss of N-CoR protein (Fig. 5C).

To further prove that lysosomes were the source of N-CoR cleaving activity presented in NSCLC cells, digestion of Flag-tagged N-CoR incubated with the extract of lysosomal or non-lysosomal fractions of H2170 cells was determined. Flag-tagged N-CoR incubated with the extract of lysosomal fraction of H2170 cells was digested completely while Flag-tagged N-CoR incubated with non-lysosomal fractions was not (Fig. 5D, left panel), suggesting that N-CoR cleaving activity was most likely localized in the lysosomes. Under identical assay condition, Flag-tagged N-CoR incubated with the lysosomal fraction of DMS-79 cells was not digested (Fig. 5D, right panel). To prove that N-CoR is indeed a substrate of CMA, a lysosomal uptake assay, in which CMA substrates taken up by chymostatin-treated lysosomes would be protected from proteinase K digestion, was performed [21]. Flag-tagged N-CoR or Hsc70, a known CMA substrate, was incubated with proteinase K and lysosomes isolaed from chymostatin treated H1270 cells, and level of N-CoR or Hsc70 proteins protected from proteinase K digestion was determined by western blotting. A significant amount of Flag-tagged N-CoR as well as Hsc70 was protected from proteinase K digestion when incubated with chymostatin treated lysosomes, suggesting that N-CoR, like Hsc70, can be translocated to the lysosomal cavity (Fig. 5E).

**Figure 6:**
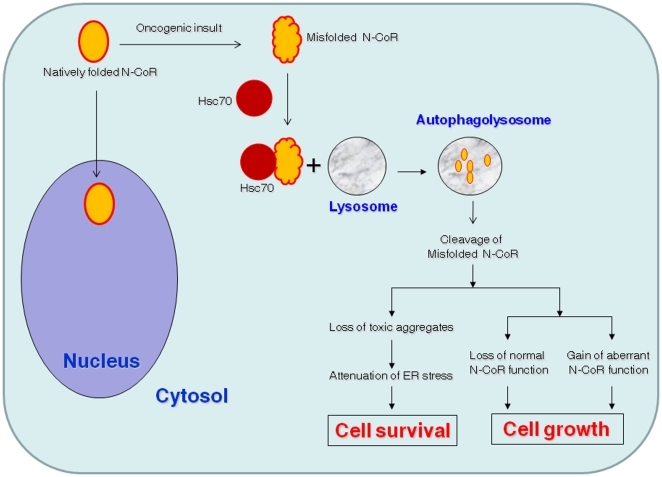
Schematic representation of the role of CMA-induced degradation of misfolded N-CoR in NSCLC cells. This model illustrates how CMA-induced degradation of misfolded N-CoR could contribute to the survival and growth of NSCLC cells through the loss of normal function and gain of aberrant function mechanism.

Based on the data described so far in this report, the potential role of CMA-induced degradation of misfolded N-CoR protein in the survival and growth of NSCLC cells is illustrated through a schematic model (Fig. 6). This model highlights how N-CoR loss could possibly contribute to the survival and growth of NSCLC cells through a dual mechanism. Degradation of misfolded N-CoR protein, on one hand, could indirectly contribute to the survival of NSCLC cells by neutralizing the ER stress caused by intracellular accumulation of misfolded N-CoR protein. At the same time, loss of N-CoR function due to misfolding may lead to the activation of oncogenic survival pathways that are normally repressed by a natively folded and functional N-CoR protein. Moreover, misfolded N-CoR and its degraded fragments could also directly activate various oncogenic mechanism linked to the growth and survival of NSCLC cells. Thus, CMA-induced degradation of misfolded N-CoR may contribute to the growth and transformation of NSCLC cells through a combination of loss and gain of function mechanisms.

## Discussion

The N-CoR instability or misfolding observed in NSCLC cells might have resulted either from a conformation altering mutation in N-CoR open reading frame that altered its natural folding landscape or by an aberrant post-translational modification that compromised its conformation. Sequence analysis of *N-CoR* open reading frame in NSCLC cell lines used in this study, however, revealed no mutation that could be attributed to its conformational change in NSCLC cells (data not shown). Whether any aberrant post-translational modification, such as phosphorylation or ubiquitination, was linked to the misfolding and instability of N-CoR in NSCLC cells is currently being investigated. Previously, we have demonstrated how PML-RAR-induced aberrant post-translational modification of N-CoR contributed to its misfolding and instability in APL cells [7], [8]. It is likely that N-CoR misfolding and instability in NSCLC cells was an outcome of similar post-translational modification triggered by unknown oncogenic events linked to the transformation of NSCLC cells. In this context, it is noteworthy that a fusion onco-protein EML4–ALK has recently been indentified and implicated in the malignant growth and transformation of NSCLC cells [32]. It would be interesting to investigate if EML4-ALK or any other NSCLC specific oncogenic events can promote PML-RAR like destabilizing effect on N-CoR conformation. Irrespective of the nature of underlying mechanism, the misfolded conformation dependent loss of N-CoR can contribute to the malignant growth and transformation of NSCLC cells through multiple mechanisms; such as loss of normal tumor suppressive function of N-CoR due to misfolding, neutralization of ER stress due to the degradation of misfolded N-CoR and possible gain of aberrant function by the misfolded N-CoR protein.

N-CoR is actively involved in the suppression of several oncogenic pathways, including PI3K/Akt/mTOR pathway which has been linked to the malignant growth and transformation of cells in NSCLC [33]–[35]. It is likely that loss of N-CoR function due to misfolding may contribute to the survival and growth of NSCLC cells through the activation of PI3K/Akt/mTOR pathway. Indeed, our recent finding demonstrated a selective activation of Akt and mTOR in NSCLC cells, and this NSCLC cell specific Akt and mTOR activation could be abrogated by genistein, the agent which restores the normal N-CoR conformation and function (Azhar and Khan et al. manuscript in preparation) [23]. In addition to the loss of normal function due to misfolding, the misfolded N-CoR or its processing by CMA may contribute to malignant growth and transformation through yet to be identified aberrant gain of function mechanism. For instance, the misfolded N-CoR or its degradation-induced fragments may actively induce oncogenic pathway linked to the growth and survival of NSCLC cells. Moreover, as demonstrated previously in APL, autophagic degradation of misfolded N-CoR may protect NSCLC cells from ER stress-induced apoptosis by attenuating misfolded N-CoR-induced ER stress. Moreover, the activation of CMA by misfolded N-CoR may represent a metabolic reprogramming of NSCLC cells which could sustain the survival and growth of tumor cells in a nutrient depleted and stressful microenvironment widely presented in solid tumors like lung cancer.

## Supporting Information

Supporting Information S1 Table 1. Lung cancer cell lines used in this study. Table 2. Primary human NSCLC derived tissue used in this study.
(PDF)
