# Supplementary material for: Role of Chaperone Mediated Autophagy (CMA) in the Degradation of Misfolded N-CoR Protein in Non-Small Cell Lung Cancer (NSCLC) Cells
Source: PLoS One. 2011 Sep 23;6(9):e25268. doi: 10.1371/journal.pone.0025268 (PMC3179509; doi:10.1371/journal.pone.0025268)
Supplement: Supporting Information S1 — Table 1. Lung cancer cell lines used in this study. Table 2. Primary human NSCLC derived tissue used in this study. (PDF) [file pone.0025268.s001.pdf]

Lung cancer cell lines used in this study

Nos.	Name of cell line	ATCC No.	Disease subtype	N-CoR protein level
1	H1299	CRL-5803	NSCLC (Large cell carcinoma)	+
2	H358	CRL-5807	NSCLC (bronchioloalveolar)	-
3	H596	HTB-178	adenosquamous carcinoma (NSCLC)	-
4	H2170	CRL-5928	squamous cell carcinoma (NSCLC)	-
5	DMS-79	CRL-2049	SCLC	+++
6	H23	CRL-5800	NSCLC	-
7	HLF-a	CCL-199	epidermoid	-
8	H647	CRL-5834	adenosquamous carcinoma (NSCLC)	-
9	H1650	CRL-5883	adenocarcinoma (NSCLC)	-
10	H1703	CRL-5889	adenocarcinoma; (NSCLC)	-
11	SW1573	CRL-2170	alveolar cell carcinoma	-
12	H1869	CRL-5900	NSCLC	+
13	H1666	CRL-5885	adenocarcinoma; bronchoalveolar carcinoma (NSCLC)	-
14	H650	CRL-5835	bronchoalverolar carcinoma; (NSCLC)	+
15	H2286	CRL-5938	adenocarcinoma; squamous cell carcimoa (NSCLC)	-

Table 1

Primary human NSCLC derived tissue used in this study

No	NT number	Disease subtype	% of tumor cells in sample
1	T01: NT00/0093	Adenocarcinoma, mixed pattern	-
2	T02: NT04/0223	Adenocarcinoma, mixed-subtype, moderate to poorly differentiated	-
3	T03: NT08/0310	Adenocarcinoma, well differentiated with mix pattern	-
4	T05: NT00/0065	Adenocarcinoma-Moderately differentiated	75
5	T03: NT07/0210	Adenocarcinoma-Moderately differentiated	70
6	T05: NT08/0482	Adenocarcinoma-poorly differentiated	70
7	T01: NT10/0004	Adenocarcinoma-Moderately differentiated	75
8	T01: NT10/0054	Adenocarcinoma-Moderately differentiated	65
9	T01: NT10/0144	Adenocarcinoma-Mixed subtype	75
10	T01: NT10/0207	Adenocarcinoma-Mix subtype mod. differentiated	70

Table 2

Supporting Legends

Table 1. The name, number and histological subtypes of cells as mentioned in the vendor's catalogues are presented. The relative level of full length N-CoR protein in various Lung cancer cells determined by western blotting was presented in column 5 and was graded by "+" or "-" symbols depending on intensity level of N-CoR protein band. Level of full length N-CoR protein in most NSCLC derived cells was very low and undetectable by western blotting with N-CoR antibody. Level of full length N-CoR protein was highest in SCLC derived cells DMS-79 (+++) and very low (-) in most NSCLC derived cells.

Table 2. The identification number, histological subtype and relative homogeneity of primary NSCLC samples obtained from the tissue repository of National University Hospital and used in this study are presented.
